# The Role of Fluoxetine in Activating Wnt/β-Catenin Signaling and Repressing β-Amyloid Production in an Alzheimer Mouse Model

**DOI:** 10.3389/fnagi.2018.00164

**Published:** 2018-06-01

**Authors:** Min Huang, Yubin Liang, Hongda Chen, Binchu Xu, Cuicui Chai, Pengfei Xing

**Affiliations:** ^1^Department of Neurology, The Seventh Affiliated Hospital, Sun Yat-sen University, Shenzhen, China; ^2^Department of Neurology, The First Affiliated Hospital of Jinan University, Guangzhou, China; ^3^Department of Traditional Chinese Medicine, The Seventh Affiliated Hospital, Sun Yat-sen University, Shenzhen, China

**Keywords:** fluoxetine, Alzheimer’s disease, Wnt/β-catenin signaling, protein phosphatases of type 2A (PP2A), amyloid-β

## Abstract

Fluoxetine (FLX) is one of the selective serotonin reuptake inhibitors (SSRIs) antidepressants, which could be used to relieve depression and anxiety among AD patients. This study was designed to search for new mechanisms by which fluoxetine could activate Wnt/β-catenin signaling pathway and reduce amyloidosis in AD brain. Fluoxetine was administered via intragastric injection to APP/tau/PS1 mouse model of Alzheimer’s disease (3×Tg-AD) mice for 4 months. In the hippocampus of AD mouse model, there could be observed neuronal apoptosis, as well as an increase in Aβ (amyloid-β) production. Moreover, there is a strong association between down-regulation of Wnt/β-catenin signaling and the alteration of AD pathology. The activity of protein phosphatases of type 2A (PP2A) could be significantly enhanced by the treatment of fluoxetine. The activation of PP2A, caused by fluoxetine, could then play a positive role in raising the level of active β-catenin, and deliver a negative impact in GSK3β activity in the hippocampal tissue. Both the changes mentioned above would lead to the activation of Wnt/β-catenin signaling. Meanwhile, fluoxetine treatment would reduce APP cleavage and Aβ generation. It could also prevent apoptosis in 3×Tg-AD primary neuronal cell, and have protective effects on neuron synapse. These findings imply that Wnt/β-catenin signaling could be a potential target outcome for AD prevention, and fluoxetine has the potential to be a promising drug in both AD prevention and treatment.

## Introduction

Alzheimer’s disease (AD) is a chronic neurodegenerative disease characterized by progressive memory decline and cognitive impairment (Scharre and Chang, 2002). AD has various histopathological hallmarks, including neurofibrillary tangles, cerebral amyloid senile plaques, synaptic and neuronal loss in the brain (Suh and Checler, 2002). Senile plaques, one of the important histopathological hallmarks, consist of a dense core of amyloid-β peptide (Aβ) and the dystrophic neuritis surrounded (Selkoe, 2004). The sequential proteolytic process of β-amyloid precursor protein (APP) through β- and γ-secretases could generate Aβ (Oulès et al., 2012). Previous study shows that Aβ could disrupt synapses and initiate a cascade of toxic events, which might result in neuronal loss (Liu et al., 2014). Another early feature of AD, besides amyloid pathogenesis, is synaptic dysfunction, which might be even prior to Aβ deposition (Miller-Thomas et al., 2016; Zuroff et al., 2017). PP2A plays a core part in dephosphorylation of inactive β-catenin and phosphorylated APP (Seeling et al., 1999; Sontag et al., 2007). Additionally, Aβ generation could also be inhabited simultaneously by drugs that target inactive PP2A (Liu and Wang, 2009; Triaca et al., 2016).

The Wnt/β-catenin signaling pathway has been found to be critical for both neuronal development and maintenance of the nervous system (Patapoutian and Reichardt, 2000). Research shows that Wnt/β-catenin signaling pathway would influence various neuronal processes, such as synaptic differentiation, synaptic function, the function of neuronal circuits, dendrite development, and neuronal plasticity (Rosso and Inestrosa, 2013). Without the activation of the Wnt/β-catenin pathway, β-catenin in the cytoplasm could be phosphorylated by a complex set of proteins, such as glycogen synthase kinase-3β (GSK3β), for ubiquitylation and degradation. However, the GSK3β activity could be inhibited by the activation of the Wnt/β-catenin pathway, which would in term lead to the repression of β-catenin phosphorylation, and, ultimately, result in the degradation of proteasome (Davidson et al., 2005; Zeng et al., 2008). Previous research shows that, in both sporadic and familial AD patients, there could be observed a decreased level of active β-catenin, inactive PP2A, and hyper active GSK3β (Voronkov et al., 2011; Inestrosa and Varela-Nallar, 2014). According to mentioned studies, it could be implied that it would be constructive and therapeutic for AD patients to maintain and rescue Wnt/β-catenin signaling.

Fluoxetine (FLX), as a selective serotonin reuptake inhibitors (SSRIs) antidepressant, could be used to relieve depression and anxiety among AD patients (Modrego, 2010). Moreover, studies suggest another potential application of FLX. For patients with mild cognitive impairment (MCI), which is a prodromal state of AD, Fluoxetine could improve the memory and cognitive function (Mowla et al., 2007). Besides, fluoxetine has been shown to be able to inhibit β-amyloid production, and prevent neuronal degeneration in an APP/PS1 mouse model (Wang et al., 2014; Ma et al., 2017; Sun et al., 2017). Furthermore, Li et al. (2004) have showed that fluoxetine could greatly enhance the phosphorylation of GSK3β. And Pilar-Cuellar et al. (2012) have revealed that fluoxetine could increase the β-catenin level. However, more research would be required to clarify if the neuroprotective effect of fluoxetine is related to the action of Wnt/β-catenin.

The purpose of our research is to explore the role of fluoxetine and its underlying mechanism in alleviating AD symptom. The research would utilize a triple-transgenic mouse model of AD, and measure the AD symptom by PP2A dependent Wnt/β-catenin signaling. Fluoxetine treatment dramatically slowing down the production of Aβ in the hippocampus of AD mouse. Moreover, during the process where fluoxetine positively influences the activation of Wnt/β-catenin signaling, promotion of PP2A activity is found to play a significant role. Ultimately, the mechanisms behind the regulation of fluoxetine in the Wnt/β-catenin signaling pathway were explored.

## Materials and Methods

### Drugs and Reagents

Fluoxetine was manufactured by Sigma-Aldrich (St. Louis, MO, United States). All cell culture reagents were produced from Invitrogen. Penicillin–streptomycin and poly-D-lysine, were manufactured by Sigma-Aldrich (St. Louis, MO, United States). Papain was obtained from Worthington. The Aβ1–42 ELISA kits and Aβ40 ELISA kits were purchased from Nanjing SenBeiJia Biological Technology Co., Ltd. (Nanjing, China). Antibodies information was in the **Table 1**. All other reagents were reagent grade.

**Table 1:** Antibody information.

Antibody	Host	Application	Source	Dilutions
Aβ1–42	Rabbit	WB/IF	Abcam	1:500
APP	Mouse	WB	Abcam	1:3000
CTFβ	Mouse	WB	Santa Cruz	1:500
CTFα	Rabbit	WB	Santa Cruz	1:500
ADAM_10_	Rabbit	WB	Abcam	1:1000
BACE1	Rabbit	WB	Abcam	1:3000
PS1	Mouse	WB	Abcam	1:1000
sAPPα	Mouse	WB	USBiological	1:1000
sAPPβ	Rabbit	WB	Abcam	1:500
C99	Rabbit	WB	Pierce	1:2000
C83	Rabbit	WB	Pierce	1:2000
BDNF	Mouse	WB	Abcam	1:1000
NeuN	Rabbit	IF	Abcam	1:300
Bcl-xL	Rabbit	WB	Abcam	1:1000
Bcl-2	Rabbit	WB	Abcam	1:1000
Cleave-Caspase3	Rabbit	WB	Cell signaling	1:1000
Bax	Rabbit	WB	Abcam	1:1000
PP2Ac	Rabbit	WB	Abcam	1:1000
PP2A pY307	Rabbit	WB	Abcam	1:1000
GSK3β pY216	Rabbit	WB	Abcam	1:1000
GSK3β	Rabbit	WB	Abcam	1:5000
Active β-catenin	Rabbit	WB/IF	Cell signaling	1:2000
Inactive β-catenin	Rabbit	WB	Cell signaling	1:2000
PSD95	Rabbit	WB/IF	Abcam	1:500
Synaptophysin	Rabbit	WB	Abcam	1:1000
GAPDH	Rabbit	WB	Abcam	1:2500

*WB, western blot blatting; IF, immunofluorescence.*

### Animals and Treatment

This study utilized 3×Tg-AD mice expressing APPswe, PS1M146V, and tauP301L human gene mutants, which were purchased from the Jackson Laboratory (Bar Harbor, ME, United States). In these mice, intracellular Aβ was detected at ages between 3 and 6 months, and cognitive impairment was detected at the age of 6 months (Oddo et al., 2003a,b; Billings et al., 2005). In the treatment group (Tg + FLX) (*n* = 12; 6 males and 6 females), fluoxetine was administrated at 10 mg/kg/day, intragastrical injection for 4 months. The fluoxetine administration began at the 4 months of age, just before they developed cognitive impairment and key pathologic features. The dose of fluoxetine was chosen aligning with previous studies (Li et al., 2009), with no gender differences. The rest two groups, the 3×Tg-AD mice group (Tg) (*n* = 12; 6 males, and 6 females) and male non-transgenic wild-type (WT) mice group (*n* = 12, 12 males), were treated with drinking water instead. All these three groups were kept under the same standard laboratory conditions with the treatment group, including temperature of 22 ± 2°C, 12-h light/dark cycle, and free access to water and food. Each cage contained 3 or 4 subjects with the same genotype. All experiments were conducted following the *Animal Care and Institutional Ethical Guidelines* in China to minimize animal suffering, for instance, reducing the number of animals used, and utilizing alternatives to *in vivo* techniques, if available. The experiments and procedures utilized in this study were conducted strictly according to the institutional guidelines regarding experimental animal use in Sun Yat-sen University. The protocol was approved by the Animal Ethical and welfare Committee of Sun Yat-sen University (Permit Number: SYXK 2016-0112).

### Behavioral Tasks

#### Morris Water Maze Test

At 8 months of age, all mice were subjected to the Morris water maze task (Mueller et al., 2008) for 5 days (d), as an evaluation of their learning and memory abilities. During the 5-day evaluation, the treatments in both experiment and control groups remained the same. All the apparatus and the test procedure utilized were described before (Vorhees and Williams, 2006). Briefly, the apparatus included a circular white metal pool, whose diameter was 160 cm and height 50 cm, and the pool was filled with 26-cm deep water at constant temperature (22 ± 1°C) throughout the experiment. The water pool was divided into four quadrants by the water maze software, and had a translucent acrylic platform. The translucent acrylic platform was 12 cm in diameter, and 24 cm in height. It was placed in the center of the northwest quadrant, 1∼2.0 cm below the water surface.

#### Spatial Learning Test

The spatial learning task was conducted for five training days with four consecutive trials per day. The location of platform was remained constant with the starting position chosen among four quadrants in sequence at the pool rim every day. At the beginning of each trial, the mice would be gently released into the water with their noses against the wall at each starting point (north, south, east, and west). Every mouse was given a maximum of 60 s to find the hidden platform. If the mouse failed to find the escape platform within 60 s on the training day, it was then manually guided to the platform for 30 s. A camera was mounted in the ceiling directly above the pool to record the escape trace of each mouse. All trials were recorded using an HVS (human visual system) water maze program for subsequent analyses of escape latency (Water Maze 3, Actimetrics, Evanston, IL, United States). All experimental procedures were performed in a blind method, where investigators were blinded to group assignment of each mouse.

#### Probe Trial

To evaluate short-term and long-term memory consolidation, probe trials were conducted at 24 and 72h, respectively, after the last trial. To perform the memory consolidation evaluation, the platform was removed at first. Then, the mice were placed into the quadrant of the pool opposite to the one pre-placed with the platform. The mice were given 60 s to swim in each probe trial. Both the time spent in the quadrant preplaced with the platform and the time spent across the platform position were recorded to evaluate short-term and long-term memory.

### Primary Culture of Hippocampal Neurons

Primary hippocampal neurons were achieved from postnatal (P0–P1) 3×Tg-AD and WT mice pups born within 24 h. After being dissected from the brain, the hippocampi were then digested with 2 mg/mL papain for 30 min at 37°C. Afterwards, the digested tissue was triturated and suspended in DMEM with 10% FBS. Dissociated cells were cultured in the neurobasal medium with 0.5 mM of L-glutamine, 2% B27 supplement, and 50 U/mL of penicillin–streptomycin. All the cultivation process would be conducted in poly-D-lysine-coated 6-well cell culture plates/culture dishes at a density of 0.5 × 10^6^ cells/per well. The cells were cultured in a 37°C incubator with 95% O_2_ and 5% CO_2_. The medium was completely replaced after 4 h, and half of the medium was then replaced every 3 days. On day 13, the neurons were treated with 1 μM fluoxetine (diluted from 20 mM fluoxetine stock solution dissolved in culture medium) for 24 h. Neurons from 3×Tg-AD mice treated with the culture medium were set up as the control group.

### ELISA for Aβ1–42 and Aβ40 Levels

Aβ1–42 and Aβ40 levels were assessed by ELISA (enzyme-linked immunosorbent assay). Primary cultured hippocampal neurons and medium from WT, 3×Tg-AD and 3×Tg-AD+fluoxetine groups were collected. The levels of extracellular and intracellular Aβ1–42 and Aβ40 were evaluated through a sandwich ELISA kit, following the manufacturer’s instruction.

### Western Blot Assay

In the brain tissue-based assay, lysis buffer plus 1 mM PMSF and protease inhibitor cocktail were utilized to homogenize brain tissue samples from different group. In the cell-based assay, the utilized cells were harvested after 24 h of 1 μM of fluoxetine treatment. Then, the harvested cells were lysed with lysis buffer. BCA protein assay kit was utilized to measure protein concentration. And SDS-PAGE was used to extract the same amount of total protein (20 μg per well) from each sample. The extracted protein would then be transferred to polyvinylidene fluoride (PVDF) membranes. After blocking with 5% fat-free milk, corresponding primary antibodies (**Table 1**) were used to probe, which would then be incubated with HRP-conjugated anti-rabbit antibody or HRP-anti-mouse antibody. The blots were developed with ECL detection reagents and visualized with a KODAK Image Station 4000 MM (Carestream Health Inc., New Haven, CT, United States). All band intensities were quantified using Quantity One software.

### Immunofluorescence Staining and Histological Analysis

Five-micrometer-thick sagittal paraffin sections of mouse hippocampus were mounted on glass slides. Before incubation, they were pretreated with 0.01 mol/L citrate buffer (pH = 6.0) in hyperthermy for 5 min. 5% goat serum in PBS was used to block the sections for 10 min. After the above pretreatments, there performed two incubations. The first one was performed with primary antibodies at 4°C, overnight. The second incubation was with secondary antibodies (1: 500 in PBS) at 37°C, 1 h. The primary antibodies used were specific to Aβ1–42, NeuN, and β-catenin. The Alexa-Fluor fluorescent dye-conjugated secondary antibodies (anti-mouse and anti-rabbit; Alexa Fluor 488 and 695, Multi Sciences Biotech) were used to detect MAP2 and PSD95. Three equidistant sections including the whole hippocampus were assessed per sample. To analyze and quantify immunoreactive areas, these sections were imaged with fluorescence microscopy (Olympus, Japan) and analyzed with Image-Pro Plus 6.0 software (Media Cybernetics).

### TdT-Mediated dUTP Nick-End Labeling

Neurons were washed three times with 0.01 MPBS, 5 min each time. 4% paraformaldehyde was used to fix the neurons for 30 min, and 0.1% Triton X-100 in 0.1% sodium citrate was used to permeate them. The TdT-mediated dUTP nick-end labeling (TUNEL) was conducted via In Situ Cell Death Detection Kit, Fluorescein (Roche), following the manufacturer’s protocols. Hoechst 33342 was used to counter stain the neuronal nuclei. Fluorescence images were obtained with an Olympus fluorescent microscope (Olympus), and TUNEL-positive cells were counted under a 20× objective.

### Quantitative RT-PCR Analysis (qRT-PCR)

TRIzol reagent (Invitrogen, Carlsbad, CA, United States) was utilized to exact the total RNA from the cells. And a qSYBR-green-containing PCR kit (Qiagen, Germantown, MD, United States) was utilized to conduct reverse transcription and qRT-PCR reactions. The fold change was determined as 2^-ΔΔC_t_^, where *C*t standard for the number of fractional cycle where the fluorescence of each sample passed the fixed threshold. All of the real-time PCR assays were performed with the Bio-Rad IQTM5 Multicolor Real-Time PCR Detection System (United States).

### Statistical Analysis

All data was in the form of mean ± SEM or mean ± SD. All statistical analysis were performed using SPSS 19.0 software (IBM SPSS Inc., Chicago, IL, United States). Two-way repeated ANOVA was used to analyze the latency of MWM test. And two-way ANOVA and Bonferroni’s *post hoc* tests were utilized to analyze the rest data. *p* < 0.05 was considered as statistically significant.

## Results

### Spatial Learning and Memory Test

The general health conditions of the 3×Tg-AD mice during the fluoxetine treatment were carefully monitored during the trial period, and no significant changes were found in the body weight of the mice before and after the trial.

**FIGURE 1:**
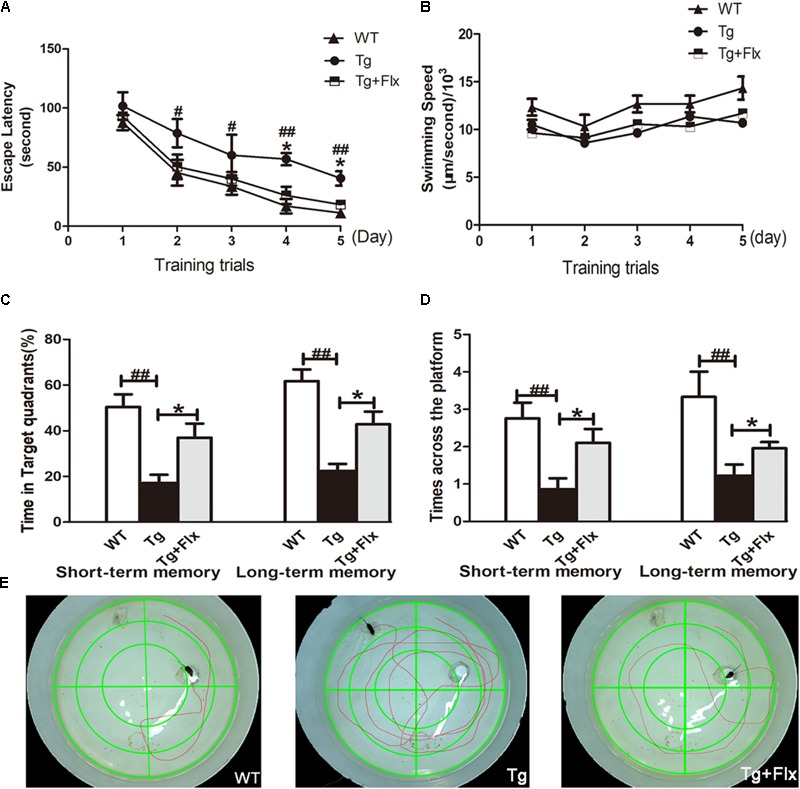
Effects of fluoxetine on spatial learning and memory of 3×Tg-AD mice detected by the Morris water maze task. **(A)** In 5-day training trials, the escaping latencies of mice were measured to evaluate the three groups mouse memory ability. Based on the results, compared with vehicle control 3×Tg-AD mice. **(B)** Regarding the swimming speed, no significant differences were observed in all groups. **(C)** In the probe trail, the frequency that mice crossed the area where the submerged platform was placed in training trials was recorded. **(D)** In terms of the time spent searching for the pre-placed platform in the target quadrant, in both the short-term and long-term memory tests. **(E)** The swimming tracks the mice in the three groups made in the water tank on the last day of the test. WT, wild-type mice; Tg, 3×Tg-AD mice; Tg + FLX, fluoxetine treated 3×Tg-AD mice. Data were shown as mean ± SD, *n* = 12 animals/group. ^#^*p* < 0.05, 3×Tg-AD group vs. WT group, ^##^*p* < 0.01, 3×Tg-AD group vs. WT group. ^∗^*p* < 0.05, fluoxetine treated 3×Tg-AD vs. 3×Tg-AD group.

Spatial learning was evaluated by the length of the time to find the hidden platform (i.e., escape latency). The results of all mice during the water maze acquisition training could be found in **Figure 1A** (*p* < 0.05). From the perspective of daily escape latency, the spatial learning ability of the mice was effectively improved in both groups after the 5-day consecutive training period. Compared with the 3×Tg-AD mice group, a significantly shorter escape latency was observed for the fluoxetine-treated 3×Tg-AD mice (*p* < 0.05). However, in *post hoc* multiple comparisons, there was no significant differences across all groups regarding swimming speed (**Figure 1B**, *p* > 0.05). The data suggests that fluoxetine could have a significant influence in attenuating spatial learning deficits in 8-month-old 3×Tg-AD mice.

Following the 5-day training, probe trials were conducted to evaluate short-term (24 h later) and long-term (72 h later) memory on the 6th and 8th day, respectively (**Figures 1C,D**). Compared with the WT mice group, 3×Tg-AD mice group exhibited a longer path distance (*p* < 0.01) across all trial sessions (**Figures 1C,D**). Between the fluoxetine -treated group and the 3×Tg-AD mice group, a statistically significant difference was observed in both short-term and long-term memory test in terms of the length of time searching for target quadrant, and searching for non-target quadrants (**Figure 1C**, *p* < 0.05). When the 3×Tg-AD mice group was compared with WT group, the former spent significantly longer time than the latter group. Based on the assessment of the length of time searching for the pre-placed platform in both short-term and long-term memory test, a statistically significant improvement was observed in the fluoxetine treated group, compared with the 3×Tg-AD mice group (**Figure 1D**, *p* < 0.05). Mice in the treated groups were gender-matched, and no significant gender-specific differences in the results were observed. Compared three groups follow tracks, the fluoxetine treated 3×Tg-AD mice group and WT group were more similar (**Figure 1E**).

### Fluoxetine Reduced the Production of Aβ in the Brains of 3×Tg-AD Mice

**FIGURE 2:**
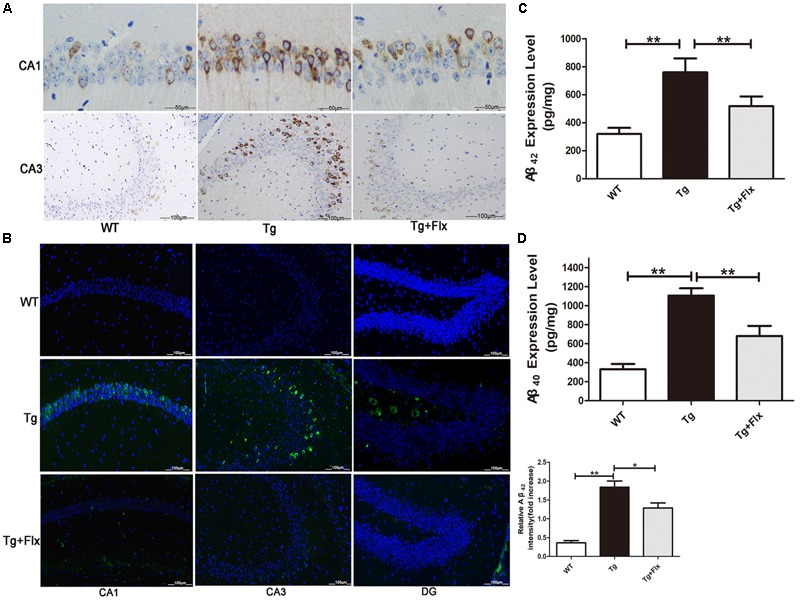
Fluoxetine reduced the production of Aβ in the brains of 3×Tg-AD mice. Four-month old 3×Tg-AD mouse were treated with vehicle or fluoxetine (10 mg/kg/day) every day for 4 months and sacrificed for analysis. **(A)** Immunohistochemical staining for β-amyloid in the hippocampus of mice from the three groups. Scale bars: 100 μm. **(B)** Immunofluorescence of Aβ42 (green) in the hippocampi of mice from the three groups. Scale bars: 100 μm. **(C,D)** The levels Aβ42 and Aβ40 in the brains of three groups were detected by ELISA. WT, wild-type mice; Tg: 3×Tg-AD mice; Tg + FLX: fluoxetine treated 3×Tg-AD mice. Data were shown as mean ± SD, *n* = 12 animals/group.^∗^*p* < 0.05, ^∗∗^*p* < 0.01.

The effect of fluoxetine on Aβ burden in the brain was also investigated in the 3×Tg-AD mice. **Figures 2A,B** indicated the results of immunofluorescent staining and immunohistochemical staining for β-amyloid in the hippocampus of mice from all three groups. While little aggregation of β-amyloid was found in the WT mice, in the other two groups there could observe β-amyloid aggregation in the hippocampus (**Figures 2A,B**). Moreover, between the 3×Tg AD group and the 3×Tg AD + Flx group, the density of β-amyloid aggregating in the hippocampus was higher in the 3×Tg AD group, and the labeling for β-amyloid was also more intense (**Figure 2B**, *p* < 0.05). These findings were validated by ELISA test. The levels of Aβ42 and Aβ40 increased significantly in the hippocampus of 3×Tg AD mice, when compared with the WT mice. Treatment with fluoxetine could inhibit the level of Aβ42 and Aβ40 in the 3×Tg AD mice (**Figures 2C,D**, *p* < 0.01).

### Fluoxetine Enhances Non-amyloidogenic Processing of APP in 3×Tg-AD Mice Brain

**FIGURE 3:**
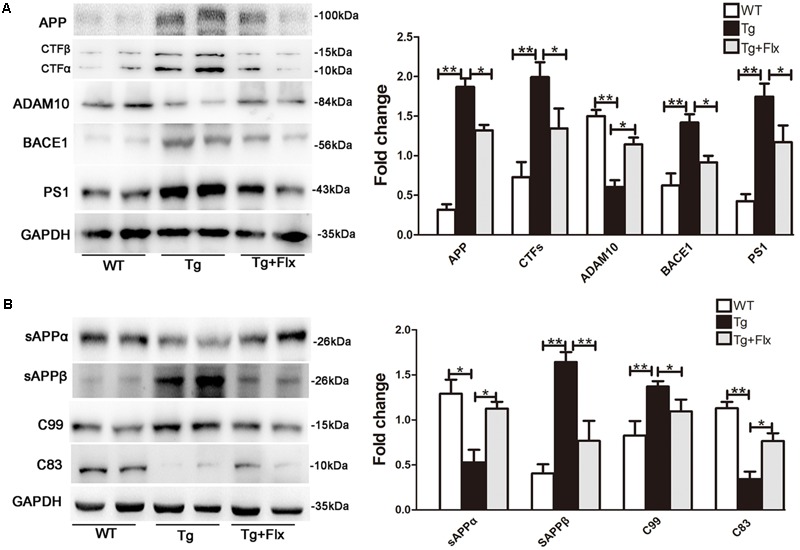
Fluoxetine enhances the non-amyloidogenic pathway in 3×Tg-AD mice brain. **(A)** The western blot analysis was used to examine the expression levels of APP and APP cleavage enzymes, including APP, CTFβ, CTFα, ADAM10, BACE1, and PS1. **(B)** Levels of APP cleavage fragments, including sAPPα, sAPPβ, C83, and C99 generation, in the brain of three groups were assessed and quantified by immunoblotting. WT, wild-type mice; Tg: 3×Tg-AD mice; Tg + FLX: fluoxetine treated 3×Tg-AD mice. All data were shown as mean ± SD, *n* = 12 animals/group. ^∗^*p* < 0.05, ^∗∗^*p* < 0.01.

To explore the impacts of fluoxetine on APP processing, the study also measured the levels of APP protein in 3×Tg-AD mouse brain with a 4-month fluoxetine treatment. As shown in **Figure 3A**, there was a significant increase in the levels of APP protein in the hippocampus of 3×Tg AD mice, compared with that of the WT mice (*p* < 0.01). Treatment with fluoxetine could reduce the level of APP protein in the 3×Tg AD mice (**Figure 3A**, *p* < 0.05). Then, it was investigated if fluoxetine was involved in APP cleavage. Western blot analysis was utilized to detect the APP cleavage enzymes and cleavage fragments in fluoxetine-treated 3×Tg-AD mouse brain (**Figure 3A**). Compared with that of WT mice, the protein levels of CTFs, BACE1, PS1, sAPPβ, and C99 in the hippocampus of 3×Tg AD mice were significantly higher. However, fluoxetine treatment could reduce the levels of protein-CTFs, BACE1, PS1, sAPPβ, and C99 in the hippocampus (**Figures 3A,B**) of the 3×Tg AD mice. We found that, compared with WT mice, 3×Tg AD mice had lower levels of ADAM10, sAPPα, and C83 in the hippocampus of, while fluoxetine treatment markedly enhanced the expression of ADAM10, sAPPα, and C83 in the hippocampus of 3×Tg AD mice.

### The Promoting Effect of Fluoxetine on BDNF in the Brain of 3×Tg-AD Mice

**FIGURE 4:**
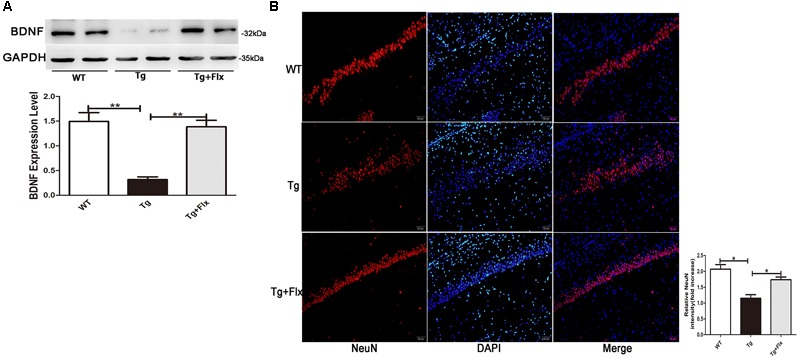
Fluoxetine promotes BDNF in the brain of 3×Tg-AD mice. **(A)** The expression levels of BDNF were examined by western blot analysis. **(B)** Immunofluorescence of NeuN (red) in the hippocampi of mice from the three groups. Scale bars: 100 μm. WT, wild-type mice; Tg, 3×Tg-AD mice; Tg + FLX, fluoxetine treated 3×Tg-AD mice. Data were shown as mean ± SD, *n* = 12 animals/group. ^∗^*p* < 0.05, ^∗∗^*p* < 0.01.

The levels of BDNF in brain of three groups mice were measured. As indicated in **Figure 4A**, 3×Tg AD mice had remarkably lower levels of BDNF protein in the hippocampus than that of the WT mice (*p* < 0.01). Treatment with fluoxetine could have a positive impact on increasing the levels of BDNF protein in the 3×Tg AD mice (*p* < 0.01). **Figure 4B** indicated the results of immunofluorescent staining for NeuN in the hippocampus of mice from three groups, there was a decrease the number of cells stained with NeuN antibody in the 3×Tg AD mice compare with WT mice (**Figure 4B**, *p* < 0.05). Treatment with fluoxetine could increase the number of cells stained with NeuN antibody in the 3×Tg AD mice (**Figure 4B**, *p* < 0.05).

### Fluoxetine Treatment Inhibited Apoptosis in Hippocampal Primary Neurons

**FIGURE 5:**
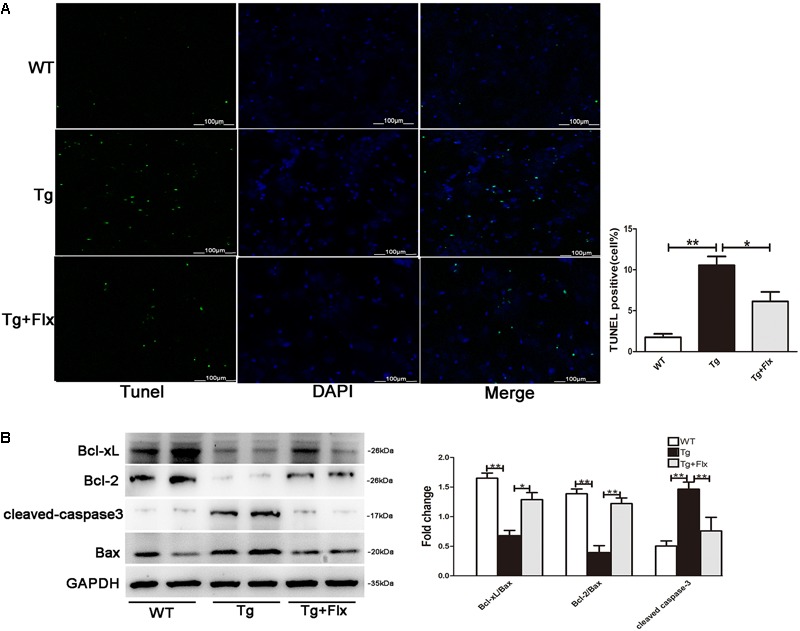
Fluoxetine treatment inhibited apoptosis in hippocampal primary neurons. **(A)** The number of TUNEL-positive neurons (green). The number of TUNEL-positive neurons per 200× field in each group (*n* = 6) was averaged. ^∗∗^*p* < 0.001 vs. control; Scale bar = 100 μm. **(B)** The western blot analysis was adopted to determine the Bcl-2, Bcl-xl cleaved-caspase 3, and Bax protein levels. WT, primarily cultured neurons of wild-type mice; Tg, primarily cultured neurons of 3×Tg-AD mice; Tg + FLX, primarily neurons of 3×Tg-AD mice treated with fluoxetine. Data were expressed as fold of control. ^∗^*p* < 0.05 compared with control. ^∗∗^*p* < 0.01 compared with control.

As shown in **Figure 5A**, the 3×Tg AD primary neurons had a larger number of TUNEL-positive cells than the WT primary neurons (*p* < 0.01). Treatment of fluoxetine at 1 μM significantly reduced the number of TUNEL-positive cells (*p* < 0.05) in the 3×Tg AD primary neurons. As proved in previous research, the ratio between Bcl-2/Bcl-xl and Bax is correlated with apoptosis (Liu et al., 2005). Thus, to investigate the molecular mechanism of the protective effect of fluoxetine in 3×Tg-AD primary neurons apoptosis, the expression of Bcl-2, Bcl-xl, and Bax was examined. Compared with the WT primary neurons, there could observe significant reduction in both Bcl-xl/Bax and Bcl-2/Bax expression ratio in the 3×Tg AD primary neurons. Treatment with fluoxetine significantly improved the Bcl-xl/Bax and Bcl-2/Bax expression ratio in the 3×Tg AD primary neurons. The levels of cleaved-caspase 3 were up-regulated in the 3×Tg AD primary neurons compared with the WT primary neurons (**Figure 5B**, *p* < 0.01). The results from the 3×Tg AD primary neurons demonstrated that treatment fluoxetine could significantly reverse this change (**Figure 5B**, *p* < 0.01).

### Fluoxetine Targeted PP2A to Activate the Wnt/β-Catenin Signaling

**FIGURE 6:**
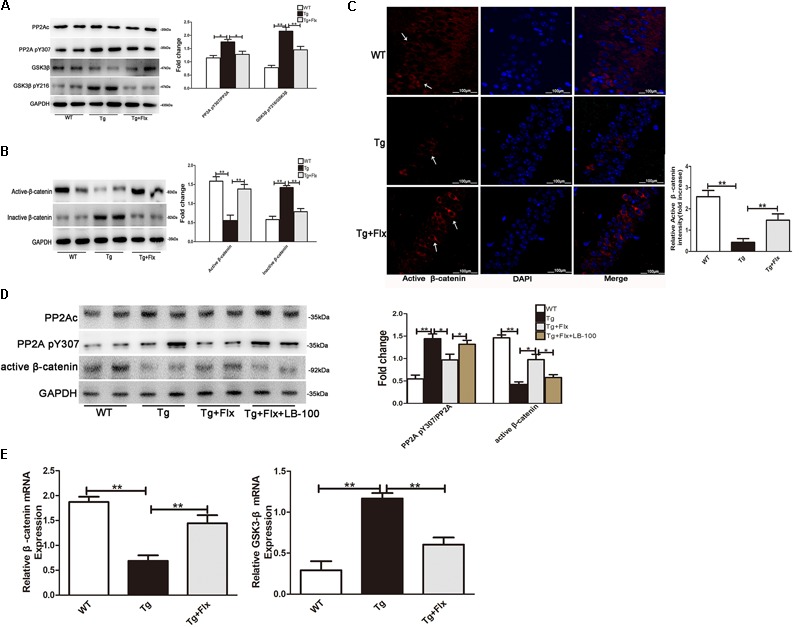
**(A)** Western blots results that showed the expression levels of GSK3β and PP2A in the brains of mice. **(B)** Active β-catenin, and inactive β-catenin were studied through Western blot. **(C)** Immunofluorescence of β-catenin (red) and DAPI (blue) in the hippocampi of mice. WT, wild-type mice; Tg, 3×Tg-AD mice; Tg + FLX, fluoxetine treated 3×Tg-AD mice. **(D)** Analysis of primarily cultured neurons from mouse hippocampus. WT, primarily cultured neurons of wild-type mice; Tg, primarily cultured neurons of 3×Tg-AD mice; Tg + FLX, primarily neurons of 3×Tg-AD mice treated with fluoxetine. Tg + FLX+LB_100, primarily neurons of 3×Tg-AD mice treated with fluoxetine and LB_100 (an inhibitor of PP2A activity). **(E)** RT-PCR showed active β-catenin and GSK3β expression in the hippocampi of mice. Data are shown as mean ± SD, *n* = 12 animals/group. ^∗^*p* < 0.05, ^∗∗^*p* < 0.01.

The activity of PP2A would be inhibited by its phosphorylation at Tyr307 (Y307) residue of PP2A catalytic subunit (PP2Ac). Inversely, the ratio of PP2A-pY307/PP2Ac could reflect the activity of PP2A *in vivo*. It was observed that this ratio was significantly higher in 3×Tg AD mice than that in the WT mice (**Figure 6A**, *p* < 0.05). This implied that treatment with fluoxetine could reduce the ratio of PP2A pY307/PP2Ac in the 3×Tg AD mice (**Figure 6A**, *p* < 0.05). As the activity of GSK3β would require Y216 phosphorylation, the ratio of GSK3β pY216/GSK3β could then imply the activity of GSK3β *in vivo*. The research provided evidence that fluoxetine could significantly decrease the ratio of GSK3β pY216/GSK3β in the 3×Tg AD mice. A significant reduction in active β-catenin was observed in the hippocampus of 3×Tg AD mice when compared with the WT mice (**Figure 6B**, *p* < 0.01). Treatment with fluoxetine could increase the active β-catenin. The hippocampus of 3×Tg AD mice also demonstrated a significant higher level of active β-catenin, compared with that of WT mice (**Figure 6B**, *p* < 0.01). Thus, treatment with fluoxetine could decrease the inactive β-catenin. It implied that fluoxetine might be sufficient to enhance active β-catenin stabilization. To further confirm the protein expression of active β-catenin in the 3×Tg-AD mice brain, an immunofluorescence analysis was performed (**Figure 6C**). Based on the immunofluorescence analysis, the level of active β-catenin in 3×Tg-AD mice was markedly increased in the treatment group (*p* < 0.01), compared with the 3×Tg-AD mice group (**Figure 6C**). Quantitative real-time (RT)-PCR analysis also confirmed that β-catenin mRNA expression was increased after fluoxetine treatment (**Figure 6D**, *p* < 0.01), while the mRNA levels of GSK3β were reduced (**Figure 6D**, *p* < 0.01) after fluoxetine treatment. The results suggested that fluoxetine treatment could efficiently activate Wnt/β-catenin signaling through inhibition of GSK3β in the 3×Tg-AD mice brain.

To further investigate if fluoxetine affected the Wnt/β-catenin signaling through PP2A activation, we isolated primarily cultured neurons, respectively, from the hippocampi of WT mouse and 3×Tg AD mouse. The cultured neurons from 3×Tg AD mouse were divided into three groups, which are 3×Tg AD, fluoxetine-treated 3×Tg AD, and fluoxetine-treated 3×Tg AD supplemented with LB-100(Tg+Flx+LB-100). LB-100, a specific inhibitor of PP2A, was used to suppress the activity of PP2A. As shown in **Figure 6E**, the 3×Tg AD primary neurons showed a significantly larger ratio of PP2A pY307/PP2Ac than the WT primary neurons. Treatment fluoxetine significantly decreased the ratio of PP2A pY307/PP2Ac in 3×Tg AD primary neurons, while adding LB-100 extensively eliminated the effect of fluoxetine. The remarkable reduction in active β-catenin between the 3×Tg AD and the WT primary neurons indicated that fluoxetine could raise the active β-catenin. Moreover, the data also demonstrated that adding LB-100 could extensively eliminate this impact from fluoxetine.

### Fluoxetine Protection of Synapses

**FIGURE 7:**
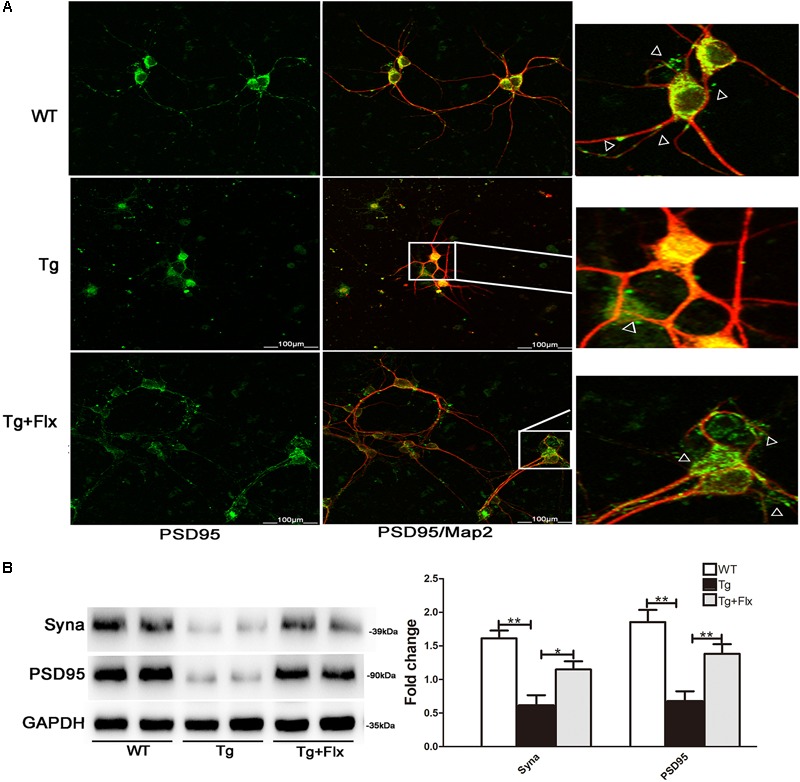
Fluoxetine protection of synapses in 3×Tg-AD neurons. **(A)** Double-labeling analysis results for neurons from WT mice, 3×Tg-AD, and fluoxetine-treated 3×Tg-AD mice. Neuronal marker MAP2 (red) with post synaptic density protein 95 (PSD95) (green). Scale bar = 100 μm. **(B)** The levels of Syna and PSD95 expression were analyzed via Western blot assay. WT, primarily cultured neurons of wild-type mice; Tg, primarily cultured neurons of 3×Tg-AD mice; Tg + FLX, primarily neurons of 3×Tg-AD mice treated with fluoxetine. Data were shown as mean ± SD, *n* = 12 animals/group. ^∗^*p* < 0.05, ^∗∗^*p* < 0.01.

To determine the impact of fluoxetine treatment on synaptic functional protein expression, immunostaining analysis was performed in primary neurons, using synaptic marker PSD95 and MAP2, as shown in **Figure 7A**. A significant reduction in PSD95 was observed in the 3×Tg-AD primary neurons when compared with the WT primary neurons (**Figure 7A**). After fluoxetine treatment, PSD95 expression was partially recovered in the neuritis of 3×Tg-AD primary neurons. In addition, western blot was used to assay the level of synaptophysin and PSD95. Compared with the culture medium 3×Tg-AD primary neurons, the treatment with fluoxetine could increase the level of both synaptophysin (**Figure 7B**, *p* < 0.05) and PSD95 (**Figure 7B**, *p* < 0.01).

## Discussion

Although fluoxetine is a well-known neuroprotective agent (Qiao et al., 2016), there exists little information about how fluoxetine influences the Wnt/β-catenin signaling in the hippocampus of AD. Moreover, it remains unconfirmed if the activation of the Wnt/β-catenin signaling pathway has any anti-AD effect.

Utilizing 3×Tg-AD mice with an early-onset AD-like pathology, the study proved that regular administration of fluoxetine could stem the age-related cognitive impairments, as well as Aβ accumulation (Oddo et al., 2003b). Our research provides supports from multiple aspects that fluoxetine could have beneficial impacts on AD. First, utilizing the 3×Tg-AD mouse model of AD, the study showed that fluoxetine could impact on brain Aβ levels, cognitive deficits, and amyloid neuropathology. Second, fluoxetine could prevent apoptosis in 3×Tg-AD primary neuronal cell. Third, fluoxetine could increase the expression of BDNF. Fourth, the study also demonstrates that fluoxetine could be able to protect neuron synapses. Moreover, our study proves evidence that fluoxetine would upregulates β-catenin expression and inhibits GSK3β expression *in vivo*. It suggests that there might be a correlation between neuroprotective effect of fluoxetine and the regulation of the Wnt signaling pathway in the AD brain.

It is widely accepted that Wnt/β-catenin signaling dysfunction plays a critical role in neurodegeneration process in the AD brain (Inestrosa and Toledo, 2008). GSK3β and β-catenin are two key components of the canonical Wnt/β-catenin signaling pathway. Previous research has demonstrated they could be considerably altered in the AD model mice brains (Zhang et al., 1998; Pei et al., 1999). Also, another study shows that activation of Wnt signaling can play a preventive impact on the neurodegeneration induced by Aβ fibrils (De Ferrari et al., 2003). The dysfunction of Wnt/β-catenin signaling induced by Aβ has been detected in AD and proved to be related to the neuron degeneration and synapse impairment. Researchers have detected the dysfunction of Wnt/β-catenin signaling induced by Aβ among AD. And this dysfunction has been proved to be associated with both neuron degeneration and synapse impairment (Thies and Bleiler, 2011; Inestrosa et al., 2012).

In this study, we showed that, when inactive β-catenin was increased in hippocampus of 3×Tg AD mice, active β-catenin could be reduced significantly. And the 3×Tg AD mouse demonstrated a remarkable reverse of these changes after treatment with fluoxetine. Meanwhile, the treatment of fluoxetine inhibited the cell apoptosis in the hippocampus of 3×Tg ADA primary neurons. These findings suggest that fluoxetine could be capable to activate the Wnt/β-catenin signaling to suppress the pathological hallmarks of AD.

Furthermore, more exploration was conducted regarding the relation across PP2A activity, Wnt/β-catenin signaling, and AD pathology, via utilizing the primarily cultured neurons from the hippocampus of 3×Tg AD mouse. Based on the results, we found that fluoxetine could significantly repair the function of Wnt/β-catenin signaling which had been impaired during the AD progression. LB-100, an inhibitor of PP2A, could extensively eliminated the effect of fluoxetine on β-catenin, which will result in the down-regulation of Wnt/β-catenin signaling and the neuronal apoptosis, Thus, it could be implied that fluoxetine could specifically activate PP2A to dephosphorylate β-catenin on S33/S37/T41 and GSK3β on Y216 for upregulation of Wnt/β-catenin signaling in 3×Tg AD mice.

Deposition of extracellular amyloid plagues in AD would also be subjected to PP2A regulation. PP2A could be capable of regulating the Aβ level through APP phosphorylation and BACE1 activity. Evidence from various aspects shows that APP could be cleaved within the sequence of the Aβ peptide and generate the sAPPα fragment through the α-secretase pathway (Esch et al., 1990). It would be beneficial for neuronal survival (Mattson et al., 1997; Wallace et al., 1997). However, through the β-secretase pathway, APP would be cleaved form neurotoxic Aβ and play a role in the pathogenesis of AD (Haass et al., 1992). This study focused on examining the role of fluoxetine in the APP process, based on the 3×Tg-AD mouse model of AD. The research found that expression level of APP proteins would be dramatically reduced in the fluoxetine-treated 3×Tg-AD mouse brain.

In this study, we examined the effects of fluoxetine on APP processing in the 3×Tg-AD mouse model of AD. Our data showed that the expression level of APP proteins would be dramatically reduced in the fluoxetine-treated 3×Tg-AD mouse brain. In this study, fluoxetine treatment could significantly enhance the expression level of ADAM10, a candidate of α-secretase. This was followed by an increase in the levels of both α-secretase-generated sAPPα and C83 fragments. Activation of the Wnt/β-catenin signaling reduced the transcription of BACE1. In our study, treatment with fluoxetine was shown to activate the Wnt/β-catenin signaling, which in term down regulated the expression of BACE1 in the hippocampus of 3×Tg AD mouse. These processes contributed to the decreased generation of Aβ. The results suggest that the Wnt/β-catenin signaling would play a significant part in regulating BACE1 expression, which then make Wnt/β-catenin signaling a core player in the formation of extracellular amyloid plagues.

Previous study has revealed that mitochondria could play a key role in regulating cell death, especially cell apoptosis (Yu et al., 2015). The dysfunction of mitochondria might become a hallmark of neuronal toxicity in AD (Xi et al., 2012; Zhang et al., 2012, 2016). Several researches also report that Bcl-2 family protein could play a certain role in regulating neuronal apoptotic cell death (Basu et al., 2006; Mancuso et al., 2008; Cartier et al., 2012). One of the key factors for the apoptotic state of cell would be the ratio between pro-apoptotic proteins and anti-apoptotic proteins (Yu et al., 2015). The result of western blot analysis shows that fluoxetine could significantly increase both expression ratios (Bcl-2/Bax and Bcl-xl/Bax) and, at the same time, decrease the number of TUNEL-positive cell compared with the culture treated with culture medium only. Thus, it is suggested that fluoxetine could inhibit the apoptosis, which involves the regulation of Bcl-2 family proteins.

BDNF is a neurotrophic factor, it is well known that BDNF is involved in the growth of neurites and synaptic plasticity (Sandhya et al., 2013). Fluoxetine might preserve synaptic protein expression, thus improve learning and memory abilities. After fluoxetine treatment, PSD95 and synaptic expression were partially recovered in the 3×Tg-AD neurons. The data above and the *in vivo* results together strongly implies that fluoxetine could be capable to abolish Aβ generation and preserve synaptic functional proteins.

## Conclusion

Treatment with fluoxetine would significantly enhance the activity of PP2A and repress the pathology of AD significantly. After activated by fluoxetine, the PP2A could increase active β-catenin level and inhibit GSK3β activity in the hippocampus of 3×Tg AD mouse, which then could lead to signal the Wnt/β-catenin signaling. The treatment could relieve the APP cleavage and Aβ generation. Taking all these results into consideration, it could be concluded that fluoxetine could activate Wnt/β-catenin signaling via PP2A to repress the cross-talk among Aβ generation and neuronal apoptosis. These results suggest that fluoxetine could have a potential for the therapy of Alzheimer’s disease through the activation of Wnt/β-catenin signaling.

## Author Contributions

MH, YL, and HC: conceived, designed, and performed the experiments. MH, YL, BX, CC, and PX: analyzed the data. MH: wrote the paper.

## Conflict of Interest Statement

The authors declare that the research was conducted in the absence of any commercial or financial relationships that could be construed as a potential conflict of interest.
